# Evaluating current practice and knowledge about antibiotic stewardship principles in paediatric tertiary hospitals to identify target areas for future teaching activities

**DOI:** 10.1007/s15010-022-01807-w

**Published:** 2022-04-02

**Authors:** Laura Kolberg, Judith Buschbeck, Annabelle Wagner, Susanne Jonat, Gerhard Wolf, Jochen Peters, Uta Behrends, Maximilian Steinhauser, Johannes Huebner, Ulrich von Both

**Affiliations:** 1 Department of Paediatric Infectious Diseases, Dr. von Hauner Children’s Hospital, Ludwig-Maximilians University, Lindwurmstr. 4, 80337 Munich, Germany; 2 Department of Paediatric Cardiology, Saarland University Medical Center, Homburg, Germany; 3 Department of Paediatrics, Clinic Starnberg, Starnberg, Germany; 4 Department of Paediatrics, Children’s Hospital Traunstein, Ludwig-Maximilians University, Munich, Germany; 5 Department of Paediatrics, Dritter Orden Clinic, Munich, Germany; 6 Department of Paediatrics, School of Medicine, Schwabing Municipal Hospital, Technical University of Munich, Munich, Germany; 7 German Center for Infection Research (DZIF) Partner Site Munich, Munich, Germany

**Keywords:** Antibiotic stewardship, ASP, Paediatrics, Germany, Training, Education

## Abstract

**Purpose:**

Antibiotic exposure among hospitalized children is very high. With inappropriate antimicrobial use resulting in increased rates of antimicrobial resistance, the implementation of antibiotic stewardship programs is critically needed. This survey study aimed to identify current practice and knowledge about antibiotic stewardship and infection control among paediatricians in tertiary care paediatric hospitals in and around Munich, Germany.

**Methods:**

A prospective cross-sectional study based on an anonymous questionnaire, structured into different sub-sections regarding antibiotic use, antimicrobial resistance, antibiotic stewardship and infection control, was conducted between 1st of May and 30th of June 2016 in five paediatric hospitals.

**Results:**

In total, 111 paediatricians across all grades were eligible for participation. The overall proportion of correct answers for all sub-sections of the survey ranged from 54.1% correct answers in the antibiotic handling and bacterial resistance section to 72.9% correct answers in the hospital hygiene/infection control section. In general, knowledge across all categories was similar for junior doctors, middle-grade doctors or consultants. Advocating empiric use of narrow-spectrum instead of broad-spectrum antibiotics was considered to be the most difficult measure to implement in daily practice (36.9%). De-escalation from broad-spectrum empirical therapy to targeted treatment was considered the easiest measure to achieve (43.2%).

**Conclusion:**

Our results demonstrate that principles of antimicrobial stewardship and aspects of hospital hygiene/infection control are not satisfactorily known among hospital-based paediatricians in and around Munich. We identified four important target areas for future educational interventions that should play a more prominent role in both pre- and postgraduate medical training.

**Supplementary Information:**

The online version contains supplementary material available at 10.1007/s15010-022-01807-w.

## Purpose

Organisms resistant to antibiotics are increasing rapidly resulting in a global public health threat. Increasing rates of antimicrobial resistance (AMR) are a direct consequence of inappropriate antimicrobial use. [1] Hospitalized children and adolescents have a very high antibiotic exposure, with 60% of them receiving at least one antibiotic per stay. [2] Furthermore, a great amount of administered antibiotics is unnecessary or inadequately prescribed. [1, 3]

Therefore, optimization of antimicrobial use and improvement towards rational prescribing of these valuable drugs is critically needed. This will result in a decrease of antibiotic resistance rates, improvement of patient care and reduced hospital stay for inpatients as well as a reduction in costs attributable to the inappropriate use of antibiotics. [4, 5] To achieve these goals antibiotic stewardship programs (ASP) with different strategies and bundle approaches for rational use of antibiotics are implemented in hospital and ambulatory care. [1, 6]

The aim of this survey study was to identify current practice and knowledge related to antibiotic stewardship aspects in paediatric junior doctors, middle-grade doctors and consultants in tertiary care paediatric hospitals in a south-eastern region in Germany using a questionnaire. The results of the survey should subsequently help to identify areas for future educational activities.

## Methods

### Setting and survey design

A prospective cross-sectional study was conducted in five German paediatric teaching hospitals in and around Munich (south-eastern region of Germany). The study was based on an anonymous questionnaire distributed among 278 active hospital-based paediatricians at the Dr. von Hauner Children’s Hospital, the children’s hospital Dritter Orden and the Children’s Hospital Schwabing as well as in two regional district general hospitals (Klinikum Traunstein and Starnberg).

### Questionnaire structure and implementation

The design of the survey was adjusted based on the work published by Bowes et al., with specific adaptations to better assess relevant areas of knowledge. [7] It was distributed between 1st of May and 30th of June 2016. Participants were able to fill in the survey via an online platform or complete a paper questionnaire that was subsequently entered into the online database—survey monkey (www.surveymonkey.de). If not stated otherwise only one answer was allowed for each question. For some questions ‘I don’t know’ was given as a possible answer option (which was considered ‘incorrect’ for the analysis). (see supplementary file 1).

The questionnaire was structured into six different sub-sections: questions regarding participants’ characteristics and education (5 questions), the handling of antibiotics and bacterial resistance (5 questions), the understanding of microbial aspects of infectious disease (4 questions), the knowledge about hospital hygiene (3 questions), antibiotic stewardship and treatment standards (7 questions) as well as questions assessing the individual respondent’s work environment (4 questions).

### Statistical analysis

Distribution of variables in the survey population was described in absolute numbers (n) and percentages (%) for junior doctors (doctors in paediatric training), middle-grade doctors (board certification, clinical registrar level) and consultants (board certification, clinical consultant level) separately. Knowledge for different sections was computed as the proportion of questions correctly answered to the total number of questions in the regarding section. The data were checked for independency using Fischer’s exact tests. The statistical software R (version 3.6.0) was used to perform the statistical analysis. [8]

## Results

### Demographics

A total of 118 participants returned the survey. Two questionnaires were answered by medical students and were therefore excluded from the analysis. Of the remaining 116 questionnaires returned by paediatricians (response rate 42%) five additional participants were excluded (one who did not state his/her position and four due to insufficient data, leaving 111 questionnaires for analysis. Of these, 47 were junior doctors, 34 were middle-grade doctors and 30 were consultants. Overall, 66/111 (59.5%) physicians were working at the Dr. von Hauner Children’s Hospital with the remaining respondents (45/111; 40.5%) distributed among other paediatric hospitals. More consultants (4; 13.3%) reported having advanced training in infectious disease, microbiology or hospital hygiene in comparison to junior doctors (*n *= 1) and middle-grade doctors (*n* = 0; *p*-value = 0.02). Two among them were working at the Dr. von Hauner Children’s Hospital and the remaining three were from other paediatric hospitals.

Overview of all questions and answers of the 111 participating paediatricians can be found in supplementary file 2.

### Antibiotic handling and bacterial resistance

The following proportion of respondents correctly answered questions regarding antibiotic prescribing and drivers of AMR: lower versus higher dosing of antibiotics, 101 (91.0%); longer versus shorter duration of therapy 77 (69.4%); use of piperacillin versus ampicillin 62 (55.9%); use of azithromycin versus clarithromycin 63 (56.8%). With regards to aspects of local data on AMR, knowledge on macrolide-resistant group A *Streptococcus* (GAS) and penicillin-resistant *Streptococcus pneumoniae* isolates yielded an astonishingly low number of correct answers (Table 1). In both cases, consultants demonstrated better knowledge (36.7 and 20.0% correct answers) compared to junior doctors (23.4 and 4.3%) and middle-grade doctors (20.6 and 2.9%). In addition, many physicians were unsure (answering “I don’t know”) about the correct answers regarding macrolide-resistant group A *Streptococcus* isolates (23/111) and penicillin-resistant *Streptococcus pneumoniae* isolates (3/111). Community carrier rate of ESBL was correctly estimated by 58 (52.3%) physicians and 57 (51.4%) identified cefotaxime as a risk factor for the selection of Clostridium difficile. In summary, the proportion of correct answers in the section of antibiotic handling and bacterial resistance was 46.5% for junior doctors, 50.7% for middle-grade doctors and 57.5% for consultants resulting in an overall mean of 54.1% (SD 25.2%) (Table 2).

**Table 1:** Knowledge of antimicrobial stewardship principles and individual opinions on implementing respective measures amongst 111 hospital-based paediatricians—selected questions and answers

	Missing (*n*)	%	*n*	%	*p*-value*
What is the current prevalence of macrolide resistance in the group A *Streptococcus* (GAS) population according to national/regional data?	1	0.9			0.36
Correct (>10%)			29	26.1	
Incorrect			81	73.0	
What is the prevalence of penicillin resistance in the *Streptococcus pneumoniae* population according to national/regional data?	0	0			**0.03**
Correct (<1%)			9	8.1	
Incorrect			102	91.9	
An inpatient with evidence of MRSA in a nasopharyngeal swab should be isolated according to which isolation scheme?	2	1.8			**0.04**
Correct (basic measures + droplet isolation)			43	38.7	
Incorrect			66	59.5	
An inpatient with pulmonary tuberculosis should be isolated according to which isolation scheme?	3	2.7			0.85
Correct (basic measures + aerogenic isolation)			82	72.9	
Incorrect			26	23.4	
Which antibiotics require therapeutic drug monitoring (TDM)?	4	3.6			0.30
Correct (vancomycin and amikacin)			37	33.3	
Incorrect			70	63.1	
Which of the following do you consider the most difficult measure to implement to improve antibiotic therapy *in your work environment*?	17	15.3			0.90
De-escalation from broad-spectrum empirical therapy to targeted treatment following receipt of pathogen differentiation and antibiogram			8	7.2	
Rapid conversion from IV to oral antibiotic therapy			7	6.3	
Reduction of therapy duration			15	13.5	
Stop antibiotic therapy in the absence of documented infection			23	20.7	
Increased use of narrow-spectrum antibiotics instead of broad-spectrum antibiotics			41	36.9	
Which of the following do you consider the easiest measure to implement to improve antibiotic therapy *in your work environment*?	15	13.5			0.75
De-escalation from broad-spectrum empirical therapy to targeted treatment following receipt of pathogen differentiation and antibiogram			48	43.2	
Rapid conversion from IV to oral antibiotic therapy			21	18.9	
Reduction of therapy duration			8	7.2	
Stop antibiotic therapy in the absence of documented infection			18	16.2	
Increased use of narrow-spectrum antibiotics instead of broad-spectrum antibiotics			1	0.9	

*Fisher’s exact test

Bold values: significant difference (*p*-value < 0.05)

**Table 2:** Overall percentage of correct answers per sub-section (mean and standard deviation, SD)

Antibiotic handling and bacterial resistance	Mean: 54.1%; SD = 25.2%
Microbial aspects of infectious disease	Mean: 56.75%; SD = 14.2%
Hospital hygiene/infection control	Mean: 72.9%, SD = 27.8%
Antibiotic stewardship and treatment standards	Mean: 55.9%; SD = 24.4%

### Microbial aspects of infectious disease

For the section regarding the microbial aspects of infectious diseases, the overall proportion of respondents identifying the correct answers was 58.1%. Answer patterns were similar for junior doctors, middle-grade doctors and consultants. The overall mean was 56.75% (SD 14.2%) (Table 2).

### Hospital hygiene/infection control

Hand hygiene was identified as the most important infection control measure by almost all 104/111 (93.7%) paediatric physicians. The appropriate isolation scheme for a patient with nasal MRSA colonization (basic measures + droplet isolation) was only reported in 43/111 (38.7%). The majority of paediatricians considered basic measures + contact isolation sufficient measures in this situation (40.5%; 45/111). Of note, consultant-level doctors showed a significantly higher rate of false answers (24/30, 80.0%) compared to middle-grade (18/34, 52.9%) or junior doctors (24/47, 51.1%; *p* value = 0.04). On the other hand, an inpatient with pulmonary tuberculosis was correctly identified as requiring aerogenic isolation measures by 82/111 (72.9%) respondents, showing no significant difference between training grades of physicians (Table 1). In summary, the proportion of correctly answered questions in the section of hospital hygiene was 70.2% for junior doctors, 72.5% for middle-grade doctors and 62.2% for consultants resulting in an overall mean of 72.9% (SD 27.8%) (Table 2).

### Antibiotic stewardship and treatment standards

Overall, the knowledge of antibiotic stewardship and treatment standards was 51.7% for junior doctors, 48.7% for middle-grade doctors and 54.8% for consultants resulting in an overall mean of 55.9% (SD 24.4%) (Table 2). Only one third of the physicians correctly identified vancomycin and amikacin as antibiotics requiring therapeutic drug monitoring (TDM) (Table 1). When sub analysing the knowledge on TDM, a significantly greater proportion of physicians was aware that vancomycin requires TDM compared to aminoglycosides (86.6 vs. 34.2%; *p*-value < 0.001). When evaluating correct empiric antibiotic choice for common clinical scenarios correct answers were provided by 16.2% (preferred antibiotic therapy for a patient with appendicitis) and 82.0% (preferred antibiotic therapy for a patient with pneumonia), respectively.

### Structure of the work environment

While consultants predominantly rely on guidelines when choosing the best antibiotic (24/30, 80.5%) only 48.9% of junior doctors and 67.6% of middle-grade doctors consult these sources. Both middle and junior grade doctors are more likely to ask for advice from their superiors or colleagues than to consult guidelines (*p* value < 0.01). Overall, 74 (66.7%) physicians were aware of and considered local resistance data and 19 (17.1%) mentioned national resistance data as most relevant for prescribing antibiotics. Of note, the most difficult measure to implement to improve antibiotic therapy was the use of narrow-spectrum antibiotics versus broad-spectrum antibiotics (36.9%), whereas the majority (43.2%) considered de-escalation from broad-spectrum empirical therapy to targeted treatment following receipt of pathogen differentiation and antibiogram as the easiest measure to achieve (Table 1).

## Discussion

This survey study aimed to identify current practice and knowledge on aspects of antibiotic use, ASP and infection control in tertiary care paediatric hospitals in and around Munich, Germany. Our analysis demonstrates an overall proportion of correct answers for all sub-sections of the survey of only just above 50%, thus elucidating the critical importance of continuing and improving educational activities covering all areas of antibiotic stewardship. In general, practice and knowledge across all categories did not differ significantly between junior doctors, middle-grade doctors or consultants. This is in line with similar studies, such as the survey of Bowes et al. assessing comparable sections in a survey published in 2014. [7] Alshengeti et al. developed and analysed the effectiveness of an online virtual patient learning module for paediatric residents about antimicrobial stewardship in 2016. A modified version of Bowes et al. survey [7] was used to measure the residents' knowledge. The overall knowledge score before the implementation of the ASP module was 58.2%, which is quite similar to the 55.0% we saw in our survey study. [9]The biggest lack of knowledge was observed regarding the local antibiotic resistance pattern. Knowledge on macrolide-resistant group A *Streptococcus* (GAS) and penicillin-resistant *Streptococcus pneumoni*ae isolates yielded an astonishingly low number of correct answers (26.1 and 8.1%, respectively). Of note, the pneumococcal local resistance rate was primarily correctly estimated by consultants (6/9; 66.7%). The vast majority (74.1%) of participating paediatricians did not know that more than 10% of group A *Streptococcus* (GAS) are resistant to macrolides and thus underestimated the issue. Only a very small minority (8.1%) was aware of the local penicillin-resistance rate (prevalence of <1%) in *Streptococcus pneumoniae*. Therefore, penicillin resistance in pneumococcus is likely to be overestimated leading to less frequent use of penicillins in common conditions such as community-acquired pneumonia (CAP). Bowes et al. found similar results in their study and pointed out, that this overestimation of antibiotic resistance levels can be an important reason for inappropriate prescribing and de-escalation strategies, resulting in increased use of broad-spectrum antibiotics. [7]

Regarding hospital hygiene and infection control, basic hygiene measures such as hand hygiene appeared to be well known and common practice across all participating hospitals. But only less than half of the participants were aware of the correct isolation scheme for a patient with MRSA colonization of the upper respiratory tract and pointed out that droplet rather than contact precautions are required in this scenario. [10] Of note, consultants did significantly worse when answering this question compared to middle-grade and junior doctors (*p* value = 0.04). Though consultant-grade doctors should certainly be aware of the correct precautions to be implemented on the ward, the middle-grade and junior doctors are practically dealing with this every day. On the other hand, for the scenario of pulmonary tuberculosis the majority of respondents provided the correct answer for the precautions required (72.9%) with no significant difference between grades of paediatricians. [11]

Two clinical scenarios were assessed in terms of empiric antibiotic choice. It is noteworthy to emphasize that while the choice of antibiotics for appendicitis was very variable, participants were rather uniformly suggesting ampicillin or amoxicillin for CAP. This reflects a direct effect of establishing and communicating internal guidelines because such a document had only recently been established for CAP at the Dr. von Hauner Children’s Hospital in early 2015, whereas no such document was available for a case of appendicitis at that time. Nevertheless, there is a national reference for appendicitis and pneumonia available in all hospitals (DGPI Handbuch [12]) Hence, this is additional practical proof of how implementing local guidelines is a very effective measure to improve rational antibiotic use. [13]

Therapeutic drug monitoring is an essential requirement when administering the glycopeptide vancomycin or aminoglycosides such as amikacin [13] and is a standard laboratory service available for all participating hospitals. Overall, only 37/111 (33.3%) paediatricians correctly identified both antibiotics as requiring TDM. Thus, our results clearly demonstrate that these drugs, though frequently used in paediatric and, in particular, in neonatal care [14], are most likely inappropriately applied and monitored putting the respective patients at risk for both an ineffective and potentially toxic therapy. Junior, middle-grade and consultant-level doctors were equally uninformed about the critical need for TDM. Still, when a paediatrician thinks about TDM, he/she is more likely to be aware of measuring serum levels when prescribing vancomycin than amikacin. There is a clear need to address TDM in targeted educational activities in the future.

When assessing the practical use of local, national or international guidance, consultants appeared to be the group most frequently turning to advice published in guidelines while junior and middle-grade doctors were more likely to directly consult their superiors when choosing an empiric or targeted antibiotic therapy. To a certain extent, this finding reflects an interesting difference between the Anglo-Saxon and the German medical system. While frequent rotation between workplaces (i.e. tertiary-care centres, district general hospitals) is an established standard in UK paediatric training, the majority of German junior and middle-grade doctors are spending their entire training period in the same hospital. Hence, inter-collegial bonds and influences of superiors, as well as an “in-house common practice” mode of action, is more common in Germany than in the UK system where NHS hospitals follow a more national guidelines-oriented and evidence-based-medicine approach (https://www.nice.org.uk/guidance; https://www.rcpch.ac.uk/resources/clinical-guidelines-evidence-reviews). However, only recently and after this survey study was conducted, a first national paediatric antibiotic stewardship guideline has been published. [13] In addition, recent years have seen more national guidelines being developed on topics such as paediatric community-acquired pneumonia. [15] A national paediatric guideline on appendicitis is currently under development (https://www.awmf.org/leitlinien/detail/anmeldung/1/ll/006-003.html). These activities clearly reflect that the importance and need for coherent and evidence-based guidance for paediatric ASP have been recognised by the responsible scientific bodies and societies.

The easiest and most difficult ASP measures to be implemented in one’s own clinical working environment were identified. While using less broad-spectrum antibiotics as part of empirical antibiotic therapy was considered the most difficult measure, de-escalating a broad empirical therapy after receiving the pathogen differentiation and antibiogram was considered the easiest measure to achieve. Of note, switching from IV to oral therapy was regarded as the second easiest measure to implement, while reducing the duration of therapy or stopping an antimicrobial in the context of missing signs for infection were identified as rather difficult. These results might be mirroring the activities of the ASP at the Dr. von Hauner Children’s Hospital since 2015 where an initial focus was laid on de-escalation of broad-spectrum antibiotic therapies in the light of microbiological results. [16] Similarly, participants in Bowes et al. survey considered discontinuation of antimicrobials in cases with no documented infection as most difficult. However, half of the trainees were in accordance with our findings and considered the empiric use of narrow-spectrum antibiotics versus broad-spectrum antibiotics as most difficult to achieve. [7] Various studies, such as Levy et al. [3], concluded that the failure of discontinuation and de-escalation of therapy was the most common reason for inappropriate antibiotic use. This is consistent across many publications and reflects the dilemma any clinician is facing when having to decide on continuation or discontinuation of antibiotics. There is an obvious need for better diagnostics and biomarkers to help in the decision making process towards de-escalation or discontinuation of antibiotic therapies. [13] Unfortunately, the highest number of missing entries was observed in this section of the questionnaire, again reflecting the dilemma illustrated above. Nevertheless, these discontinuation and de-escalation aspects need to be a clear focus of future educational activities of local ASPs.

Given that at the Dr. von Hauner Children’s Hospital an ASP was initiated in 2015, the results of this survey were rather sobering. [16] There was no significant difference in the knowledge of ASP measures between the Dr. von Hauner Children’s Hospital and other participating institutions.

This may be due to the rather short period of time that the ASP at the Dr. von Hauner Children’s Hospital had been in place. Furthermore, the ASP was not focused on teaching individual doctors but rather consisted of infectious diseases consultation service, development and provision of internal guidelines on empiric antibiotic therapy and clinical ward rounds of an antibiotic stewardship team formulating recommendations to assist paediatric colleagues on ward. [16] While this approach improves the quality of patient care, it may not have the same effect on the knowledge of antimicrobial stewardship principles amongst paediatricians. Thus, the implementation of a structured teaching program would be a key measure to address this aspect of improving knowledge on the individual doctor’s level.

Our results indicate that the knowledge of hospital-based paediatricians of the south-eastern region of Germany regarding the different areas of ASP is only moderate and clearly needs further improvement to optimise the clinical care in paediatric hospitals.

In contrast to the Anglo-Saxon medical system where infectious diseases training and ASP aspects have long been integrated into the medical training curricula, starting at the medical student’s level only a very small minority (5/111, 5.6%) of all clinicians participating in our study had previous training in the respective areas. Specialist training in this area is an established component of medical postgraduate training in other European countries, such as the UK, or in the USA. Unfortunately, Germany does not yet have an equivalent focus on training in infectious disease, antibiotic stewardship and hospital hygiene. Thus, the results of this study strongly suggest this area as a critical focus for university and post graduate training in paediatrics. Only sustainable educational efforts in ASP and infectious diseases will be able to tackle this evident lack of knowledge in almost all areas covered by our survey and to help us improve patient care in the years and decades to come. That ASP training improves the knowledge was shown in a recent publication analysing knowledge scores of Canadian residents. A significantly higher knowledge score (71.6%) was found four months after implementation of an ASP module compared to before the pre-implementation period (58.2%). [9]

Our study has several obvious limitations, some of which are due to the simple fact that this was only a survey study. Since we analysed data of German paediatric physicians in and around Munich (south-eastern region of Germany) and the response rate was quite low (42%) our results are not fully generalizable and may not be representative for other paediatric hospitals. We could not perform a non-responder analysis and therefore we do not know to what extent the non-responders differ from the participating clinicians.

## Conclusion

Assessment of current practice and knowledge about antimicrobial stewardship principles and aspects of hospital hygiene/infection control amongst 111 hospital-based paediatricians in and around Munich (south-eastern region of Germany) has yielded a rather unsatisfactory result. None of the areas assessed scored above 75% when evaluating the accuracy of answers to topic-related questions; three areas rather revealed an overall score of <60%. Thus, we have identified four important target areas for future educational interventions that should be given a more prominent role to play in both pre- as well as postgraduate medical training.

## Supplementary Information

Below is the link to the electronic supplementary material.

Supplementary file1 (PDF 626 KB)

Supplementary file2 (PDF 570 KB)
