# Supplementary material for: Evaluating current practice and knowledge about antibiotic stewardship principles in paediatric tertiary hospitals to identify target areas for future teaching activities
Source: Infection. 2022 Apr 2;50(5):1273–9. doi: 10.1007/s15010-022-01807-w (PMC9522681; doi:10.1007/s15010-022-01807-w)
Supplement: Supplementary file 1 — Supplementary file1 (PDF 626 KB) [file 15010_2022_1807_MOESM1_ESM.pdf]

Supplementary Information 1

Journal name: Infection

Evaluating current practice and knowledge about antibiotic stewardship principles in paediatric tertiary hospitals to identify target areas for future teaching activities

Laura Kolberg*, Judith Buschbeck, Annabelle Wagner, Susanne Jonat, Gerhard Wolf, Jochen Peters, Uta Behrends, Maximilian Steinhauser, Johannes Huebner, Ulrich von Both

*Corresponding author:

Laura Kolberg, MSc (ORCID: 0000-0002-3311-8254)

Department of Paediatric Infectious Diseases, Dr von Hauner Children's Hospital, Ludwig-Maximilians University, Lindwurmstr. 4, 80337 Munich, Germany, phone: +49 89 4400 53157, email: laura.kolberg@med.uni-muenchen.de

Questionnaire „Antibiotic Stewardship“

Basic aspects and education of participant

1 Where do you work as a paediatrician?
- a Dr. von Hauner Children’s Hospital
- b children hospital Dritter Orden
- c Children’s Hospital Schwabing
- d Hospital Starnberg
- e Hospital Traunstein

2 What is your professional role?
- a consultant
- b middle grade doctor
- c junior doctor
- d medical student

3 How often do you prescribe antibiotics per day?
- a 0-1 x/day
- b 2-4 x/ day
- c >4 x/ day

4 Have you treated patients with infection caused by antibiotic-resistant pathogens (e.g. ESBL, MRSA, VRE, 3MRGN or 4 MRGN) within the last year?
- a Yes
- b No
- c I don't know

5 Did you ever participate in any advanced training course in infectious diseases, microbiology or infection control / hospital epidemiology?
- a Yes
- b No

Handling of antibiotics and bacterial resistance

6 <u>What of the following options, do you think, contributes most to antimicrobial resistance?</u>

6.1a **low dosing**
b high dosing

6.2a **long duration of therapy**
b short duration of therapy (< 7 days)

6.3a prescription of ampicillin
b **prescription of piperacillin**

6.4a **prescription of azithromycin**
b prescription of clarithromycin

7 <u>What is the current prevalence of macrolide resistance in the group A streptococcus (GAS) population according to national / regional data?</u>

a < 1 %
b 1 - 5 %
c 5 - 10 %
d **> 10 %**
e I don't know

8 <u>What is the prevalence of penicillin resistance in the *Streptococcus pneumoniae* population according to national / regional data?</u>

a **< 1 %**
b 1 - 5 %
c 5 - 10 %
d > 10 %
e I don't know

9 <u>What, do you think, is the community carrier rate of ESBL?</u>

a 0 %
b 0-5%
c **5-10%**
d 10-20%
e >20%

10 <u>Which of the following antibiotics, do you think, contributes most to an increased risk for *Clostridium difficile* infection?</u>

a **Cefotaxime**
b Penicillin G
c Azithromycin
d Vancomycin
e I don't know

Microbial aspects of infectious disease

11 Smaller minimal inhibition concentration (MIC) values are…

- a **better**
- b worse
- c I don’t know

12 *Haemophilus influenzae* is…

- a gram-positive
- b **gram-negative**
- c I don’t know

13 After what incubation time can a result be considered "negative" in the vast majority of cases (>90%) if there is no growth in the blood culture?

- a After 1 day
- b **2-3 days**
- c 4-5 days
- d After 1 week
- e After 10 days

14 You get a call from the bacteriology lab informing you that *P. aeruginosa* **3MRGN** has grown in your patient's blood culture. Which of the following antibiotic classes does not play a role in this resistance assessment and nomenclature?

- a 3rd and 4th Generation Cephalosporine
- b Carbapeneme
- c **Aminoglykoside**
- d Fluorchinolone
- e Penicillins with extended activity spectrum e.g. piperacillin

Hospital hygiene

15 What is the most important infection control measure?

- a consequent screening of all patients for colonization
- b consequent Isolation measures
- c **strict adherence to hand hygiene**
- d consequent disinfection of patient surroundings
- e I don’t know

16 An inpatient with evidence of MRSA in a nasopharyngeal swab should be isolated according to which isolation scheme?

- a No isolation, basic hospital hygiene measures
- b Basic measures + contact isolation
- c **Basic measures + droplet isolation**
- d Basic measures + aerogenic isolation

17 An inpatient with pulmonary tuberculosis should be isolated according to which isolation scheme?

- a No isolation, basic hospital hygiene measures
- b Basic measures + contact isolation
- c Basic measures + droplet isolation
- d **Basic measures + aerogenic isolation**

Antibiotic stewardship and treatment standards

18 Which antibiotics require therapeutic drug monitoring (TDM)?
(More than 1 answer is possible)
- a Meropenem
- b **Vancomycin**
- c **Amikacin**
- d Linezolid
- e I don't know

19 You are asked to prescribe perioperative antibiotic prophylaxis for a healthy teenager who is going to have an elective neurosurgical procedure on the spine and spinal cord. Which antibiotic would you prescribe in this case?
- a Carbapenem (e.g. Meropenem) IV
- b **1st or 2nd generation Cephalosporine (e.g. Cefazolin or Cefuroxime) IV**
- c Piperacillin/tazobactam IV
- d Vancomycin IV
- e No prophylaxis

20 If you recommend perioperative prophylaxis, for how long?
- a **Preoperative single dose**
- b max. continuation of antibiotic administration until 24 h post-op
- c Continuation of antibiotic administration > 24 h post-op
- d I don't know

21 A 3-year-old child with all STIKO-recommended vaccinations gets admitted. The child presents with cough, fever and chest pain for about 4 days. The temperature is 38.5 °C, respiratory rate 30/min, heart rate 90/min and oxygen saturation in air is 98%. The chest X-ray shows an infiltrate of moderate size in the right middle lobe. Which antibiotic with the lowest possible activity spectrum would be indicated in this case?
- a a makrolid (e.g. clarithromycin) PO
- b 2nd generation Cephalosporin (e.g. cefuroxime) IV
- c **Ampicillin or penicillin IV**
- d Cefuroxime and clindamycin IV
- e I don't know

22 The child mentioned above shows clinical improvement (fever resolution after 48 hours and good appetite) and blood cultures are negative. What would you prescribe to continue oral therapy on an outpatient basis?
- a Cefuroxime PO
- b **Amoxicillin PO**
- c Amoxicillin and clavulanic acid PO
- d Clarithromycin PO
- e I don't know

23 What is the recommended therapy duration in this case of above mentioned uncomplicated bacterial pneumonia in childhood?
- a 5 days
- b **7 days**
- c 10 days
- d 14 days
- e I don't know

24 <u>You are asked to prescribe antibiotic therapy for a previously healthy 11-year-old girl who is now on her way to surgery with signs of clinical appendicitis. Which of the following antibiotics would be your first choice?</u>

- a Cefotaxime und metronidazol IV
- b Cefotaxime monotherapy IV
- c Gentamicin and clindamycin IV
- d Gentamicin and metronidazol IV
- e **Amoxicillin and clavulanic acid IV**
- f I don't know

Structure of work environment

25 <u>When deciding on antibiotic therapy for a patient, who or what do you turn to first if you have questions?</u>

- a junior doctor colleagues on ward
- b Consultant
- c Pharmacists/hospital pharmacists
- d Guidelines (e.g. Hauner AntibiotiCard, Sanford Guide, Red Book, Blue Book, DGPI manual)
- e other source, please specify:______________________________

26 <u>When prescribing antibiotics, which type of bacterial resistance data do you consider most relevant?</u>

- a local resistance data
- b national resistance data
- c global resistance reports
- d other, please specify:_________________________________
- e none

27 <u>Which of the following do you consider the **most difficult** measure to implement to improve antibiotic therapy *in your work environment*?</u>

- a Stop antibiotic therapy in absence of documented infection
- b Reduction of therapy duration
- c Rapid conversion from IV to PO antibiotic therapy
- d De-escalation from broad-spectrum empirical therapy to targeted treatment following receipt of pathogen differentiation and antibiogram
- e Increased use of narrow-spectrum antibiotics instead of broad-spectrum antibiotics

28 <u>Which of the following do you consider the **easiest** measure to implement to improve antibiotic therapy *in your work environment*?</u>

- a Stop antibiotic therapy in absence of documented infection
- b Reduction of therapy duration
- c Rapid conversion from IV to PO antibiotic therapy
- d De-escalation from broad-spectrum empirical therapy to targeted treatment following receipt of pathogen differentiation and antibiogram
- e Increased use of narrow-spectrum antibiotics instead of broad-spectrum antibiotics
