# Supplementary material for: Evaluating current practice and knowledge about antibiotic stewardship principles in paediatric tertiary hospitals to identify target areas for future teaching activities
Source: Infection. 2022 Apr 2;50(5):1273–9. doi: 10.1007/s15010-022-01807-w (PMC9522681; doi:10.1007/s15010-022-01807-w)
Supplement: Supplementary file 2 — Supplementary file2 (PDF 570 KB) [file 15010_2022_1807_MOESM2_ESM.pdf]

Supplementary Information 2

Journal name: Infection

Evaluating current practice and knowledge about antibiotic stewardship principles in paediatric tertiary hospitals to identify target areas for future teaching activities

Laura Kolberg*, Judith Buschbeck, Annabelle Wagner, Susanne Jonat, Gerhard Wolf, Jochen Peters, Uta Behrends, Maximilian Steinhauser, Johannes Huebner, Ulrich von Both

*Corresponding author:

Laura Kolberg, MSc (ORCID: 0000-0002-3311-8254)

Department of Paediatric Infectious Diseases, Dr von Hauner Children's Hospital, Ludwig-Maximilians University, Lindwurmstr. 4, 80337 Munich, Germany, phone: +49 89 4400 53157, email: laura.kolberg@med.uni-muenchen.de

Overview of all questions and answers of the 111 participating paediatricians				
	Missing			
	n	%	n	%
**Basic aspects and education of participant**				
1. Where do you work as a paediatrician?	0	0		
Dr. von Hauner Children's Hospital			66	59.5
Other			45	40.5
2. What is your professional role?	0	0		
junior doctor			47	
middle grade doctor			34	
consultant			30	
3. How often do you prescribe antibiotics per day?	0	0		
0-1 x/day			65	58.6
2-4 x/ day			39	35.1
>4 x/ day			7	6.3
4. Have you treated patients with infection caused by antibiotic-resistant pathogens (e.g. ESBL, MRSA, VRE, 3MRGN or 4 MRGN) within the last year?	0	0		
Yes			96	86.5
No			12	10.8
I don't know			3	2.7
5. Did you ever participate in any advanced training course in infectious diseases, microbiology or infection control / hospital epidemiology?	0	0		
Yes			5	4.5
No			106	95.5
**Handling of antibiotics and bacterial resistance**				
6. What of the following options, do you think, contributes most to antimicrobial resistance?				
6.1	4	3.6		
**low dosing** (correct)			101	91.0
high dosing			6	5.4
6.2	4	3.6		
**long duration of therapy** (correct)			77	69.4
short duration of therapy (< 7 days)			30	27.0

6.3	7	6.3		
**prescription of piperacillin** (correct)			62	55.9
prescription of ampicillin			42	37.8
6.4	7	6.3		
**prescription of azithromycin** (correct)			63	56.8
prescription of clarithromycin			41	36.9
7. What is the current prevalence of macrolide resistance in the group A streptococcus (GAS) population according to national / regional data?	1	0.9		
correct **(> 10 %)**			29	26.1
incorrect			81	73.0
8. What is the prevalence of penicillin resistance in the *Streptococcus pneumoniae* population according to national / regional data?	0	0		
correct (**< 1 %**)			9	8.1
incorrect			102	91.9
9. What, do you think, is the community carrier rate of ESBL?	0	0		
correct (**5-10%)**			58	52.3
incorrect			53	47.7
10. Which of the following antibiotics, do you think, contributes most to an increased risk for *Clostridium difficile* infection?	0	0		
correct **(Cefotaxime)**			57	51.4
incorrect			54	48.6
**Microbial aspects of infectious disease**				
11. Smaller minimal inhibition concentration (MIC) values are…	0	0		
correct **(better)**			92	82.9
incorrect			19	17.1
12. *Haemophilus influenzae* is…	0	0		
correct **(gram-negative)**			83	74.8
incorrect			28	25.2
13. After what incubation time can a result be considered "negative" in the vast majority of cases (>90%) if there is no growth in the blood culture?	1	0.9		
correct **(2-3 days)**			43	38.7
incorrect			67	60.4
14. You get a call from the bacteriology lab informing you that *P. aeruginosa* **3MRGN** has grown in your patient's blood culture. Which of the following antibiotic classes does not play a role in this resistance assessment and nomenclature?	2	1.8		
correct **(Aminoglykoside)**			40	36.0
Incorrect			69	62.2
**Hospital hygiene**				
15. What is the most important infection control measure?	3	2.7		
correct (**strict adherence to hand hygiene**)			104	93.7
incorrect			4	3.6
16. An inpatient with evidence of MRSA in a nasopharyngeal swab should be isolated according to which isolation scheme?	2	1.8		
correct **(Basic measures + droplet isolation**)			43	38.7
incorrect			66	59.5
17. An inpatient with pulmonary tuberculosis should be isolated according to which isolation scheme?	3	2.7		
correct **(Basic measures + aerogenic isolation)**			82	72.9
incorrect			26	23.4

**Antibiotic stewardship and treatment standards**				
18. Which antibiotics require therapeutic drug monitoring (TDM)? *(More than 1 answer is possible)*	4	3.6		
correct (**Vancomycin and amikacin**)			37	33.3
incorrect			70	63.1
19. You are asked to prescribe perioperative antibiotic prophylaxis for a healthy teenager who is going to have an elective neurosurgical procedure on the spine and spinal cord. Which antibiotic would you prescribe in this case?	4	3.6		
correct **(1st or 2nd generation Cephalosporine (e.g. Cefazolin or Cefuroxime) IV)**			62	55.9
incorrect			45	40.5
20. If you recommend perioperative prophylaxis (n=106), for how long?	5	4.5		
correct **(Preoperative single dose)**			36	38.2
incorrect			64	61.8
21. A 3-year-old child with all STIKO-recommended vaccinations gets admitted. The child presents with cough, fever and chest pain for about 4 days. The temperature is 38.5 °C, respiratory rate 30/min, heart rate 90/min and oxygen saturation in air is 98%. The chest X-ray shows an infiltrate of moderate size in the right middle lobe. Which antibiotic with the lowest possible activity spectrum would be indicated in this case?	7	6.3		
correct **(Ampicillin or penicillin IV)**			91	82.0
incorrect			13	11.7
22. The child mentioned above shows clinical improvement (fever resolution after 48 hours and good appetite) and blood cultures are negative. What would you prescribe to continue oral therapy on an outpatient basis?	6	5.4		
correct **(Amoxicillin PO)**			80	72.1
incorrect			5	22.5
23. What is the recommended therapy duration in this case of above mentioned uncomplicated bacterial pneumonia in childhood?	7	6.3		
correct **(7 days)**			77	69.4
incorrect			27	24.3
24. You are asked to prescribe antibiotic therapy for a previously healthy 11-year-old girl who is now on her way to surgery with signs of clinical appendicitis. Which of the following antibiotics would be your first choice?	10	9		
correct (**Amoxicillin and clavulanic acid IV**)			18	16.2
incorrect			83	74.8
**Structure of work environment**				
25. When deciding on antibiotic therapy for a patient, who or what do you turn to first if you have questions?	16	14.4		
junior doctor colleagues on ward or Consultant			23	20.7
Guidelines (e.g. Hauner AntibiotiKarte, Sanford Guide, Red Book, Blue Book, DGPI manual)			70	63.1
other source			2	1.8
26. When prescribing antibiotics, which type of bacterial resistance data do you consider most relevant?	11	9.9		
none			2	1.8
local resistance data			74	66.7
national resistance data			19	17.1
global resistance reports			4	3.6
other			1	0.9

27. Which of the following do you consider the **most difficult** measure to implement to improve antibiotic therapy *in your work environment*?	17	15.3		
De-escalation from broad-spectrum empirical therapy to targeted treatment following receipt of pathogen differentiation and antibiogram			8	7.2
Rapid conversion from IV to PO antibiotic therapy			7	6.3
Reduction of therapy duration			15	13.5
Stop antibiotic therapy in absence of documented infection			23	20.7
Increased use of narrow-spectrum antibiotics instead of broad-spectrum antibiotics			41	36.9
28. Which of the following do you consider the **easiest** measure to implement to improve antibiotic therapy *in your work environment*?	15	13.5		
De-escalation from broad-spectrum empirical therapy to targeted treatment following receipt of pathogen differentiation and antibiogram			48	43.2
Rapid conversion from IV to P antibiotic therapy			21	18.9
Reduction of therapy duration			8	7.2
Stop antibiotic therapy in absence of documented infection			18	16.2
Increased use of narrow-spectrum antibiotics instead of broad-spectrum antibiotics			1	0.9
